# Novel gene rearrangement in the mitochondrial genome of *Siliqua minima* (Bivalvia, Adapedonta) and phylogenetic implications for Imparidentia

**DOI:** 10.1371/journal.pone.0249446

**Published:** 2021-04-06

**Authors:** Jiantong Feng, Yahong Guo, Chengrui Yan, Yingying Ye, Xiaojun Yan, Jiji Li, Kaida Xu, Baoying Guo, Zhenming Lü

**Affiliations:** 1 National Engineering Research Center for Marine Aquaculture, Zhejiang Ocean University, Zhoushan, China; 2 National Engineering Laboratory of Marine Germplasm Resources Exploration and Utilization, Zhejiang Ocean University, Zhoushan, China; 3 Scientific Observing and Experimental Station of Fishery Resources for Key Fishing Grounds, MOA, Key Laboratory of Sustainable Utilization of Technology Research, Marine Fisheries Research Institute of Zhejiang, Zhoushan, China; Natural History Museum of London, UNITED KINGDOM

## Abstract

*Siliqua minima* (Gmelin, 1791) is an important economic shellfish species belonging to the family Pharidae. To date, the complete mitochondrial genome of only one species in this family (*Sinonovacula constricta*) has been sequenced. Research on the Pharidae family is very limited; to improve the evolution of this bivalve family, we sequenced the complete mitochondrial genome of *S*. *minima* by next-generation sequencing. The genome is 17,064 bp in length, consisting of 12 protein-coding genes (PCGs), 22 transfer RNA genes (tRNA), and two ribosomal RNA genes (rRNA). From the rearrangement analysis of bivalves, we found that the gene sequences of bivalves greatly variable among species, and with closer genetic relationship, the more consistent of the gene arrangement is higher among the species. Moreover, according to the gene arrangement of seven species from Adapedonta, we found that gene rearrangement among families is particularly obvious, while the gene order within families is relatively conservative. The phylogenetic analysis between species of the superorder Imparidentia using 12 conserved PCGs. The *S*. *minima* mitogenome was provided and will improve the phylogenetic resolution of Pharidae species.

## Introduction

The mitochondrial DNA (mtDNA) of metazoans is generally a closed circular molecule and is the only extranuclear genome in animal cytoplasm [1]. It contains its own genetic system, with maternal inheritance, low intermolecular recombination, high copy number and high substitution rate [2]. In general, mitochondrial DNA of Bivalvia contains 22 transfer RNA genes (tRNA), two ribosomal RNA genes (rRNA), 12 protein-coding genes (PCGs) and a noncoding control region, i.e., the origin of light-strand replication (OL) region [3, 4]. Complete mitochondrial genomes have become popular for phylogenetic reconstruction of animal relationships [5–8]. Molecular analysis is the most commonly method to identify species without morphological classification, which is accuracy and provides a lot of information [9]. In recent years, there were many studies on gene rearrangements and phylogenetic analysis of bivalves using the mitochondrial genomes [10–12].

The superorder Imparidentia is a newly defined branch of bivalves in 2014 [13]. Through the Paleozoic and Mesozoic, it showed stable diversification [13]. This superorder includes most marine bivalve families [14], and is of great significance in phylogenetic analysis of Bivalvia. Phylogenetics of bivalves has been a hot topic since ancient times, but there are still many deficiencies in previous studies, among which the analysis of Imparidentia yet has numerous uncertainties [15]. Encompassing Combosch et al. had conducted the systematics of the Imparidentia in the Bivalvia based on Sanger-sequencing approach, nevertheless, it is difficult to resolve the relationships within Imparidentia using this approach. Thus, they suggested that transcriptomic analysis of Imparidentia to resolve its position of a taxa [16]. Subsequently, Lemer et al. analyzed the phylogeny of Imparidentia through transcriptome data, and the Imparidentia puzzle in phylogeny was solved by establishing a data matrix optimized for Imparidentia [14]. Due to it is a new clade of definition, existing analysis is superficial, it is extremely necessary for taxonomic and phylogenetic in-depth investigate of the superorder clams.

The razor clams (e.g., Pharidae, Solenidae) are ecologically and economically important shellfish in the coastal areas of China. They are distributed in the tropics and temperate zones [17]. The family Pharidae is dominated by marine species, belonging to the order Adapedonta of Bivalvia, except for a single typically freshwater genus, *Novaculina* [18, 19]. The family Solenidae is once considered to include the family Pharidae by some authorities [20]. *Siliqua minima* (Gmelin, 1791), belong to the family Pharidae, which lives in the benthic environment from intertidal mudflats at a water depth of more than 30 m [17, 21]. It is mainly distributed in the coastal areas in the south of Zhejiang Province in China. *Siliqua minima* mainly feeds on plankton and organic debris in seawater through filtration [22]. It has gained attention because of it is ecologically and economically important in the coastal regions of China with high commercial and nutritional value [19, 23]. Previous studies of *S*. *minima* mainly focused on nutritional value evaluation, the composition and changes of fatty acids, and the effects of various environmental factors [22–24]. There are few researches on molecular level about it.

In the present study, we sequenced the first complete mitogenome of *S*. *minima* to gain insights into its adaptive evolution and study the characteristics of its mitogenomes, including nucleotide composition, codon usage and secondary structure of tRNAs. Furthermore, we performed phylogenetic analysis of the 12 protein-coding genes (PCGs) (except *atp8*) in the *S*. *minima* mitogenome with the PCGs of 54 complete mitogenomes of the superorder Imparidentia retrieved from GenBank of NCBI in order to understand its evolutionary relationship. We also integrated the gene arrangement of mitogenomes during evolution in Adapedonta in order to obtain a more accurate evolutionary relationship. These results will help to view the phylogenetic relationship of *S*. *minima* in bivalve species.

## Materials and methods

### Ethics statement

The study was conducted in accordance with the guidelines and regulations of the government. No endangered or protected species were involved. There is no special permission for this kind of razor clam, which is very common in the aquatic market. Sampling also did not require specific permissions for the location.

### Sample collection and DNA extraction

*Siliqua minima* samples were collected in November 2018 from the coastal area of Xiapu County (E120°24.8577′, N26°93.0578′), Fujian Province, in the South China Sea. Preliminary morphological identification of the specimens was carried out through the published taxonomic books [25], and a taxonomist from the Marine Biological Museum of Zhejiang Ocean University was consulted [26]. The field-collected samples were initially placed in absolute ethyl alcohol and stored at -20°C prior DNA extraction. The total genomic DNA was extracted from adductor muscle using the rapid salting-out method [27]. The quality of DNA was detected by 1% agarose gel electrophoresis, and the DNA was stored at -20°C before sequencing.

### Sequencing, assembly, and annotation of mitochondrial genomes

Complete mitogenome sequencing of *S*. *minima* was performed on an Illumina HiSeq X Ten platform (Shanghai Origingene Bio-pharm Technology Co., Ltd., China), and an Illumina PE Library of 400 bp was constructed. Quality control, de novo assembly, functional annotation and molecular evolution analysis of the *S*. *minima* mitogenome were conducted based on bioinformatics analysis methods. The NCBI has established a large database SRA (Sequence Read Archive, https://trace.ncbi.nlm.nih.gov/Traces/sra/) to store and share original high-throughput sequencing data. Clean data without sequencing adapters were assembled de novo using NOVOPlasty software (https://github.com/ndierckx/NOVOPlasty) [28]. To ensure the accuracy of the species and the correctness of sequence, we compared the mitochondrial genomes of the assembled *S*. *minima*, and used NCBI BLAST to detect the *cox1* barcode sequence for taxonomical identification [29]. The new mitogenome was annotated using the MITOS Web Server with the invertebrate genetic code (http://mitos2.bioinf.uni-leipzig.de/index.py) and then compared with its existing relatives to determine the number of genes and the position of its initial and terminal codons [30, 31].

### Genome visualization and comparative analysis

The circular map of the *S*. *minima* mitochondrial genome was generated by using the online server CGView (http://stothard.afns.ualberta.ca/cgview_server/index.html) [32]. The secondary structures of tRNAs were predicted initially by using MITOS WebServer, as well as tRNAscan-SE v.2.0 Webserver (http://lowelab.ucsc.edu/tRNAscan-SE/), and ARWEN (http://130.235.244.92/ARWEN/) was used to re-identify the numbers of tRNAs and secondary structures [33, 34]. The putative origin of L-strand replication (OL) was identified by the Mfold Web Server and edited in Adobe Photoshop CC [35]. Base composition and relative synonymous codon usage (RSCU) for 12 PCGs of *S*. *minima* were calculated and sorted using MEGA 7.0 [36]. The skew value denotes strand asymmetry, which was calculated according to the following formulas: AT skew = (A − T)/(A + T) and GC skew = (G − C)/(G + C) [37].

### Phylogenetic analysis and gene order

The software DAMBE 5.3.19 was used to quickly identify 12 PCGs in the mitochondrial genome [38]. To investigate the phylogenetic relationship of Pharidae, 54 individuals belonging to seventeen families of five orders of the superorder Imparidentia were downloaded from the NCBI. Mitogenomes of *Argopecten irradians* and *Mimachlamys senatoria* of the family Pectinidae of Pteriomorph were used as outgroups. The ClustalW algorithm in MEGA 7.0 was used to align the 12 PCGs of each species via the default settings [36]. Subsequently, to reconstruct the phylogenetic tree, the result of the multiple sequence alignment was used for the phylogenetic analysis based on the maximum likelihood (ML) and Bayesian inference (BI). The ML tree was constructed in IQ-TREE using the TVM+F+R8 model with 1000 nonparametric bootstrapping replicates and the best-fit substitution model with ModelFinder [39, 40]. Bayesian inference (BI) methods were used with the program MrBayes v3.2 [41]. By associating PAUP 4.0, Modeltest 3.7 and MrModeltest 2.3 software in MrMTgui, the best-fit model (GTR+I+G) of substitution was chosen according to AIC [42, 43]. BI analyses were conducted with Markov Chain Monte Carlo (MCMC) sampled every 1,000 generations each with three heated chains and one cold chain run for 2,000,000 generations, and the first 25% burn-in was discarded. Visualization of the tree was realized using FigTree v1.4.3 [44].

## Results and discussion

### Genome organization and base composition

**Fig 1:**
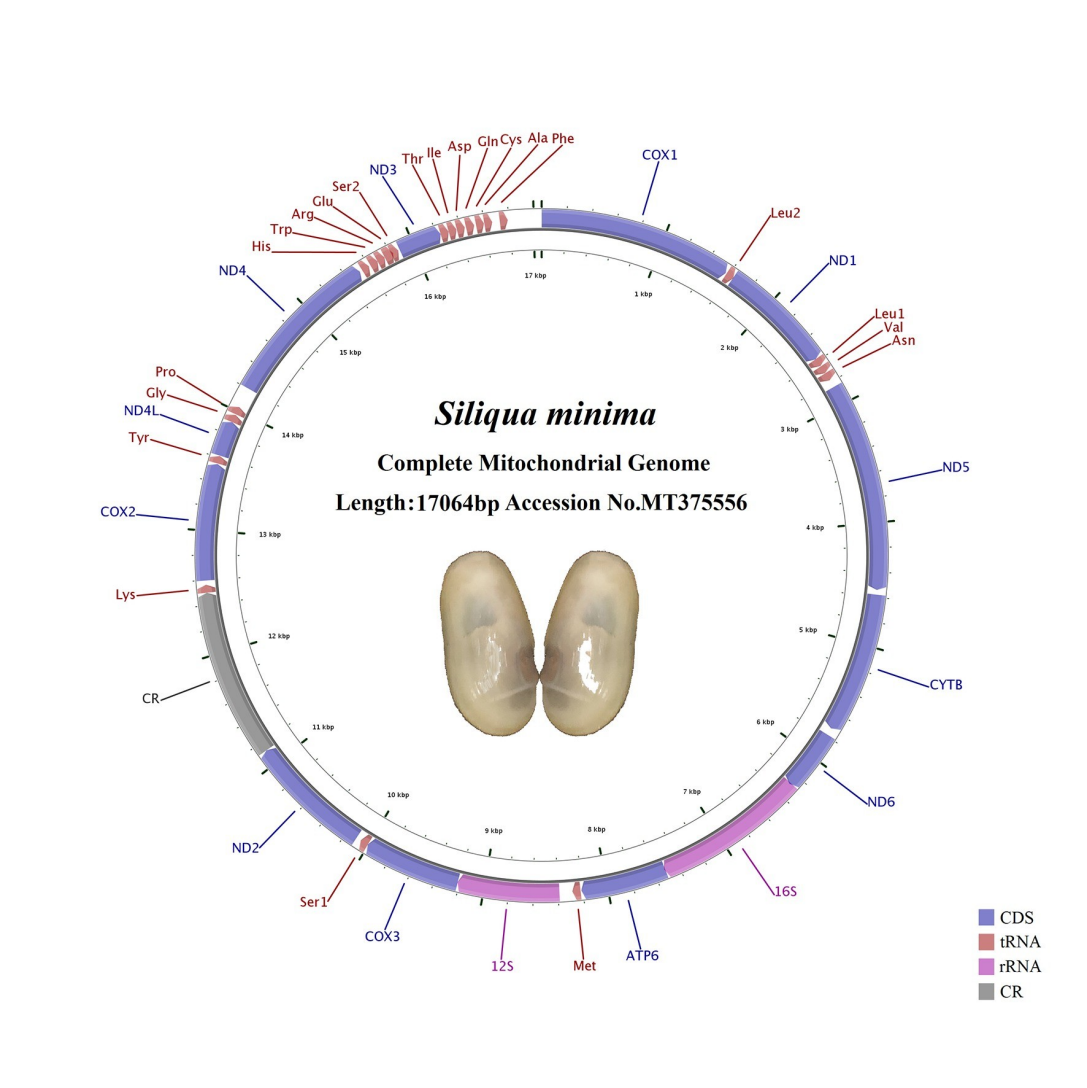
Maps of the mitochondrial genomes of *Siliqua minima*. Direction of gene transcription is indicated by the arrows.

The complete mitogenome of *S*. *minima* was 17,064 bp in length, which has been deposited in GenBank under accession NO. MT375556 (Fig 1, Table 1). In the present study, there was only one referential species, *S*. *constricta*, of Pharidae, whose mitogenome was 17,225 bp in length, similar to that of *S*. *minima* [45]. *S*. *constricta* was previously classified as belonging to the family Solecurtidae but has been confirmed to belong to the family Pharidae [31, 46]. The mitogenomes of Pharidae were longer than other species in Adapedonta observed, i.e. typically ranged from 15,381 bp (*Panopea abrupta*) to 16,784 bp (*Solen grandis*) (Table 1). The circular mitochondrial genome of *S*. *minima* had 22 putative tRNA genes, 2 rRNA genes (12S rRNA and 16S rRNA), 12 PCGs and one control region (CR) including an origin of the light-strand replication (OL) region. According to our statistics, all species of Adapedonta we downloaded contained the *atp8* gene, except for species of the family Pharidae [45, 47–51]. The gene arrangement of the mitogenome of *S*. *minima* was identical to that of *S*. *constricta*. Interestingly, all 36 mitochondrial genes were encoded on the heavy chain.

**Table 1:** List of species analysed in this study with their GenBank accession numbers.

Order	Family	Species	Size (bp)	Accession no.
Venerida	Veneridae	*Paphia amabilis*	19629	NC_016889
		*Paphia euglypta*	18643	GU269271
		*Paphia textile*	18561	NC_016890
		*Paphia undulata*	18154	NC_016891
		*Macridiscus melanaegis*	20738	NC_045870
		*Macridiscus multifarius*	20171	NC_045888
		*Dosinia japonica*	17693	MF401432
		*Dosinia troscheli*	17229	NC_037917
		*Dosinia altior*	17536	NC_037916
		*Mercenaria mercenaria*	18365	MN233789
		*Meretrix meretrix*	19826	GQ463598
		*Meretrix petechialis*	19567	EU145977
		*Meretrix lusoria*	20268	GQ903339
		*Meretrix lamarckii*	21209	NC_016174
		*Meretrix lyrate*	21625	NC_022924
		*Saxidomus purpuratus*	19637	NC_026728
		*Cyclina sinensis*	21799	KU097333
	Vesicomyidae	*Calyptogena marissinica*	17374	NC_044766
		*Calyptogena extenta*	16106	MF981085
		*Archivesica gigas*	15674	MF959623
		*Pliocardia ponderosa*	16275	MF981084
	Arcticidae	*Arctica islandica*	18289	KF363951
	Corbiculidae	*Corbicula fluminea*	17423	NC_046410
	Mactridae	*Lutraria maxima*	17082	NC_036766
		*Lutraria rhynchaena*	16927	NC_023384
		*Pseudocardium sachalinense*	17978	MG431821
		*Coelomactra antiquata*	17384	JN692486
		*Mactra chinensis*	17285	NC_025510
Cardiida	Cardiidae	*Acanthocardia tuberculata*	16104	DQ632743
		*Cerastoderma edule*	14947	NC_035728
		*Fulvia mutica*	19110	NC_022194
		*Vasticardium flavum*	16596	MK783266
	Tridacnidae	*Tridacna crocea*	19157	MK249738
		*Tridacna squamosa*	20930	NC_026558
		*Tridacna derasa*	20760	NC_039945
		*Hippopus hippopus*	22463	MG722975
	Donacidae	*Donax semiestriatus*	17044	NC_035984
		*Donax vittatus*	17070	NC_035987
		*Donax trunculus*	17365	NC_035985
		*Donax variegatus*	17195	NC_035986
	Psammobiidae	*Soletellina diphos*	16352	NC_018372
	Solecurtidae	*Solecurtus divaricatus*	16749	NC_018376
	Tellinidae	*Moerella iridescens*	16799	JN398362
	Semelidae	*Semele scabra*	17117	JN398365
Adapedonta	Hiatellidae	*Panopea abrupta*	15381	NC_033538
		*Panopea generosa*	15585	NC_025635
		*Panopea globosa*	15469	NC_025636
	Pharidae	***Siliqua minima***	17064	MT375556
		*Sinonovacula constricta*	17225	EU880278
	Solenidae	*Solen grandis*	16784	HQ703012
		*Solen strictus*	16535	NC_017616
Myoida	Myidae	*Mya arenaria*	17947	NC_024738
Lucinida	Lucinidae	*Loripes lacteus*	17321	EF043341
		*Lucinella divaricata*	18940	EF043342

The overall base composition of the whole mitochondrial genome was 25.41% A, 41.00% T, 22.93% G, and 10.62% C, exhibiting obvious AT bias (66.41%). Due to the skewness of the *S*. *minima* mitogenome, most of them are negative, except the CR and rRNAs possessed an opposite AT skew compared with other genes (Table 2). All GC-skews were positive, indicating that the base composition ratios were G biased to C.

**Table 2:** Skewness of the *S*. *minima* mitogenome.

Region	Size(bp)	A (%)	T (%)	G (%)	C (%)	A+T (%)	AT-skew	GC-skew
Mitogenome	17,064	25.41	41.00	22.93	10.62	66.41	-0.235	0.367
*cox1*	1569	20.59	44.04	22.56	12.81	64.63	-0.363	0.276
*nad1*	939	18.74	46.01	25.45	9.80	64.75	-0.421	0.444
*nad5*	1698	21.85	46.11	22.08	9.95	67.96	-0.357	0.379
*cytb*	1146	21.90	44.68	20.94	12.48	66.58	-0.342	0.253
*nad6*	501	26.75	45.11	21.36	6.79	71.86	-0.255	0.518
*atp6*	699	22.46	46.64	19.46	11.44	69.10	-0.350	0.260
*cox3*	789	21.93	42.71	22.31	13.05	64.64	-0.321	0.262
*nad2*	1017	22.91	44.05	22.91	10.13	66.96	-0.316	0.387
*cox2*	948	27.11	34.81	27.85	10.23	61.92	-0.124	0.463
*nad4l*	288	21.53	44.10	27.78	6.60	65.63	-0.344	0.616
*nad4*	1314	21.31	46.35	23.67	8.68	67.66	-0.370	0.463
*nad3*	354	17.80	48.31	26.55	7.34	66.11	-0.462	0.567
CR	1371	32.31	27.79	29.76	10.14	60.10	0.075	0.492
tRNAs	1452	30.51	35.61	21.28	12.60	66.12	-0.077	0.256
rRNAs	2076	34.97	33.86	18.98	12.19	68.83	0.016	0.218
PCGs	11,262	22.02	44.33	23.17	10.49	66.35	-0.336	0.377

### Noncoding regions and gene overlapping

Generally, the mitogenome contains a non-coding region (NR), including AT-rich, hairpin structures, tandem repeats and some peculiar patterns [52–54]. It is supposed to play a role in the regulation of mitochondrial transcription and replication [55]. There were 25 NRs in *S*. *minima*, which is similar to *S*. *constricta* (25 NR) of the same family as in previous reports [45]. The largest NR of *S*. *minima* was identified as a putative control region (CR). In addition, the longest intergenic region of the razor clam was 273 bp and was located between *trnF* and *cox1* (Table 3).

**Table 3:** Annotation of the *S*. *minima* mitochondrial genome.

Gene	Strand	Location		Length	Codons	Intergenic nucleotide (bp)	Anticodon
		Start	Stop				
*cox1*	+	1	1569	1569	ATG/TAA	8	
*trnL2*	+	1578	1643	66		-3	TAA
*nad1*	+	1641	2579	939	ATA/TAA	-1	
*trnL1*	+	2579	2645	67		4	TAG
*trnV*	+	2650	2714	65		1	TAC
*trnN*	+	2716	2782	67		63	GTT
*nad5*	+	2846	4543	1698	ATT/TAA	38	
*cytb*	+	4582	5727	1146	ATG/TAA	58	
*nad6*	+	5786	6286	501	ATT/TAG	-11	
*rrnL*	+	6276	7522	1247		-9	
*atp6*	+	7514	8212	699	ATG/TAG	6	
*trnM*	+	8219	8285	67		104	CAT
*rrnS*	+	8390	9218	829		-2	
*cox3*	+	9217	10,005	789	ATG/TAG	-1	
*trnS1*	+	10,005	10,071	67		39	TCT
*nad2*	+	10,111	11,127	1017	ATT/TAA	1371	
*trnK*	+	12,499	12,563	65		34	TTT
*cox2*	+	12,598	13,282	685	ATG/T(AA)	267	
*trnY*	+	13,550	13,612	63		3	GTA
*nad4l*	+	13,616	13,903	288	ATG/TAA	-1	
*trnG*	+	13,903	13,967	65		5	TCC
*trnP*	+	13,973	14,037	65		166	TGG
*nad4*	+	14,204	15,517	1314	ATG/TAA	21	
*trnH*	+	15,539	15,601	63		14	GTG
*trnW*	+	15,616	15,681	66		0	TCA
*trnR*	+	15,682	15,746	65		1	TCG
*trnE*	+	15,748	15,823	76		-16	TTC
*trnS2*	+	15,808	15,870	63		12	TGA
*nad3*	+	15,883	16,236	354	ATA/TAG	0	
*trnT*	+	16237	16,304	68		0	TGT
*trnI*	+	16,305	16,369	65		3	GAT
*trnD*	+	16,373	16,438	66		10	GTC
*trnQ*	+	16,449	16,516	68		15	TTG
*trnC*	+	16,532	16,596	65		3	GCA
*trnA*	+	16,600	16,664	65		61	TGC
*trnF*	+	16,726	16,790	65		273	GAA

**Fig 2:**
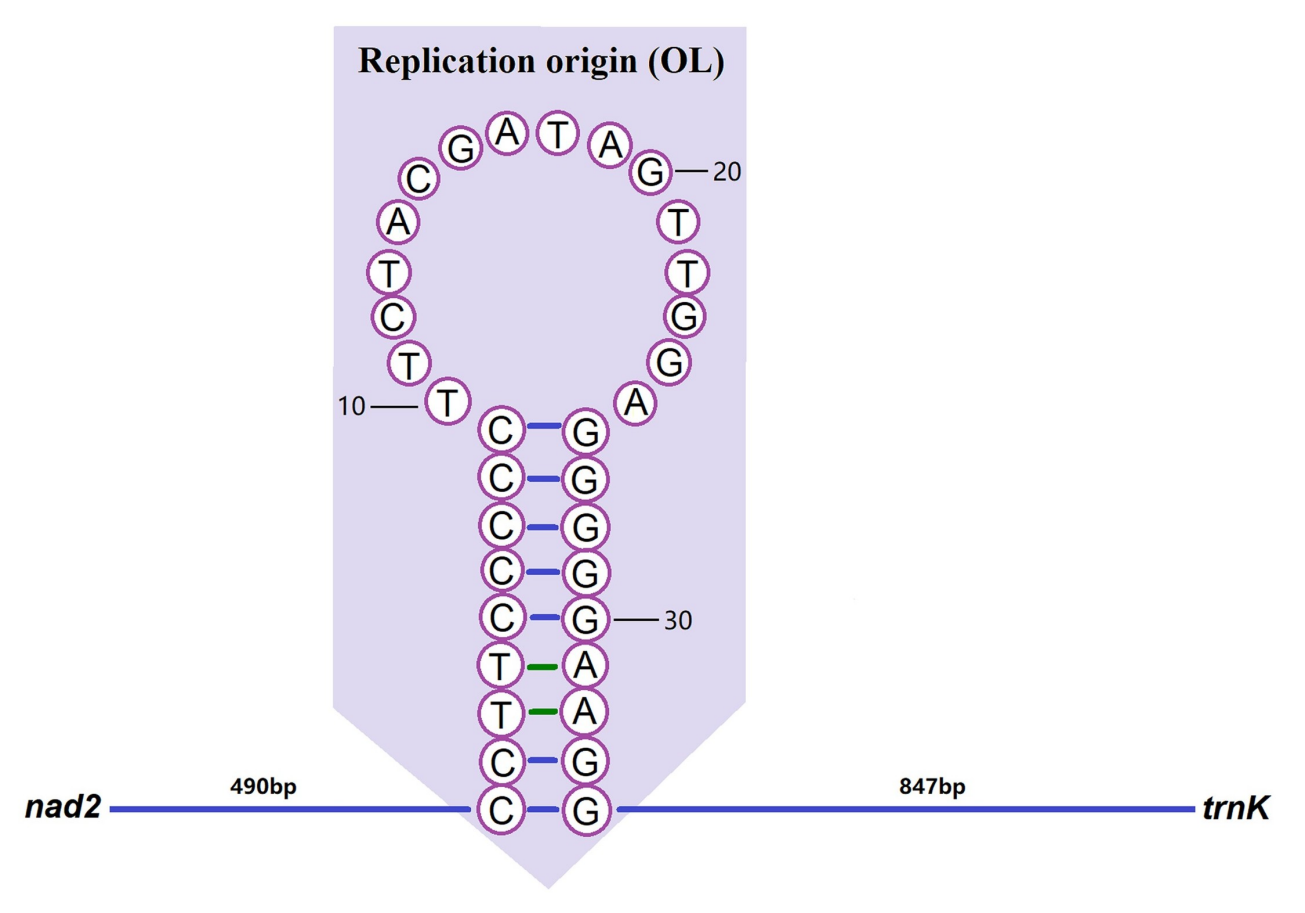
Secondary structure of the origin of L-strand replication (OL). The figure was edited in Adobe Photoshop CC.

The CR is the region with the largest length variation in the whole mitochondrial sequence and the region with the fastest evolution in the mitochondrial genome [56]. It has a high mutation rate, so it is of great value to study for population genetic analyses [57]. By comparing the gene order of bivalves, we can see that the CR regions are not conservative but are highly rearranged. The CR region was located between *nad2* and *trnK* in the *S*. *minima* mitogenome, spanning 1,371 bp with 60.10% A+T content and showing positive AT- and GC-skew (0.075 and 0.492), indicating bias towards A and G (Fig 1; Tables 2 and 3). Simultaneously, the replication origin of the L-strand (OL) region was also found in this region. The “OL” region could form a stem-loop secondary structure with 18 bp in the stem and 16 bp in the loop, with an overall length of 34 bp (CCTTCCCCCTTCTACGATAGTTGGAGGGGGAAGG), and the secondary structure of the stem-loop, which has the potential to fold, was predicted (Fig 2).

The overlapping of neighbouring genes is common in bivalve mollusc mitochondria. There were eight overlaps of neighbouring genes in the mitochondrial genome of *S*. *minima*. The position of the largest gene overlap (16 bp) was between *trnS2* and *trnE*.

### Protein-coding genes and codon usage

*Siliqua minima* has 12 PCGs and lacks the *atp8* gene, which is very common in bivalves. The total length of the 12 concatenated protein-coding genes was 11,262 bp, accounting for approximately 66.00% of the whole mitogenome (Table 2). The average A+T content was 66.35%, ranging from 61.92% (*cox2*) to 71.86% (*nad6*) (Table 2). We further compared the PCGs of the six Adapedonta species mitogenomes, and the PCGs ranged from 61.78% (*Solen strictus*) to 66.45% (*S*. *constricta*) (Table 4). The AT-skew values were negative (-0.336) for PCGs, while the GC-skew values (0.377) were positive (Table 2).

**Table 4:** Nucleotide composition in regions of the mitogenomes of seven Adapedonta species.

Species	Total size	Complete mitogenome						
		A	T	G	C	A + T%	AT-skew	GC-skew
*Panopea abrupta*	15,381	25.60	38.78	24.27	11.34	64.38	-0.205	0.363
*Panopea generosa*	15,585	25.05	38.70	25.03	11.22	63.75	-0.214	0.381
*Panopea globosa*	15,469	23.32	40.39	26.14	10.15	63.71	-0.268	0.441
***Siliqua minima***	17,064	25.41	41.00	22.93	10.62	66.41	-0.235	0.367
*Sinonovacula constricta*	17,225	25.94	41.11	22.48	10.47	67.05	-0.226	0.365
*Solen grandis*	16,784	22.62	42.22	24.51	10.65	64.84	-0.302	0.394
*Solen strictus*	16,535	21.74	40.95	25.64	11.67	62.69	-0.306	0.374
		**PCGs**						
*Panopea abrupta*	11,025	22.93	40.39	25.10	11.58	63.32	-0.276	0.369
*Panopea generosa*	11,025	22.47	40.27	25.89	11.37	62.74	-0.284	0.390
*Panopea globosa*	11,031	20.48	42.59	26.83	10.10	63.07	-0.351	0.453
***Siliqua minima***	11,262	22.02	44.33	23.17	10.49	66.35	-0.336	0.377
*Sinonovacula constricta*	11,005	22.51	43.94	22.87	10.68	66.45	-0.323	0.363
*Solen grandis*	11,526	19.19	44.97	25.37	10.47	64.16	-0.402	0.416
*Solen strictus*	11,550	18.23	43.55	26.68	11.55	61.78	-0.410	0.396
		**tRNAs**						
*Panopea abrupta*	1419	32.77	35.24	20.23	11.77	68.01	-0.036	0.264
*Panopea generosa*	1432	32.19	35.41	21.23	11.17	67.60	-0.048	0.310
*Panopea globosa*	1432	30.73	35.27	22.63	11.38	66.00	-0.069	0.331
***Siliqua minima***	1452	30.51	35.61	21.28	12.60	66.12	-0.077	0.256
*Sinonovacula constricta*	1442	30.44	35.44	21.22	12.90	65.88	-0.076	0.244
*Solen grandis*	1584	28.72	37.19	22.41	11.68	65.91	-0.129	0.315
*Solen strictus*	1444	27.49	35.18	23.96	13.37	62.67	-0.123	0.284
		**rRNAs**						
*Panopea abrupta*	1917	34.06	34.12	20.29	11.53	68.18	-0.001	0.275
*Panopea generosa*	2092	34.23	34.32	20.27	11.19	68.55	-0.001	0.289
*Panopea globosa*	2103	33.24	34.33	21.40	11.03	67.57	-0.016	0.320
***Siliqua minima***	2076	34.97	33.86	18.98	12.19	68.83	0.016	0.218
*Sinonovacula constricta*	2069	32.96	35.23	20.15	11.65	68.20	-0.033	0.267
*Solen grandis*	2185	31.72	34.97	22.38	10.94	66.69	-0.049	0.343
*Solen strictus*	2196	31.79	34.15	22.40	11.66	65.94	-0.036	0.315

For all 12 PCGs identified in the *S*. *minima* mitogenome, two genes (*nad1* and *nad3*) were initiated with the start codon ATA, three genes (*nad5*, *nad6* and *nad2*) started with the codon ATT, and the remaining seven genes had the start codon ATG. The *nad6*, *atp6*, *cox3* and *nad3* genes had the termination codon TAG (Table 3). Moreover, the most common termination codon, TAA, was detected in eight PCGs.

**Fig 3:**
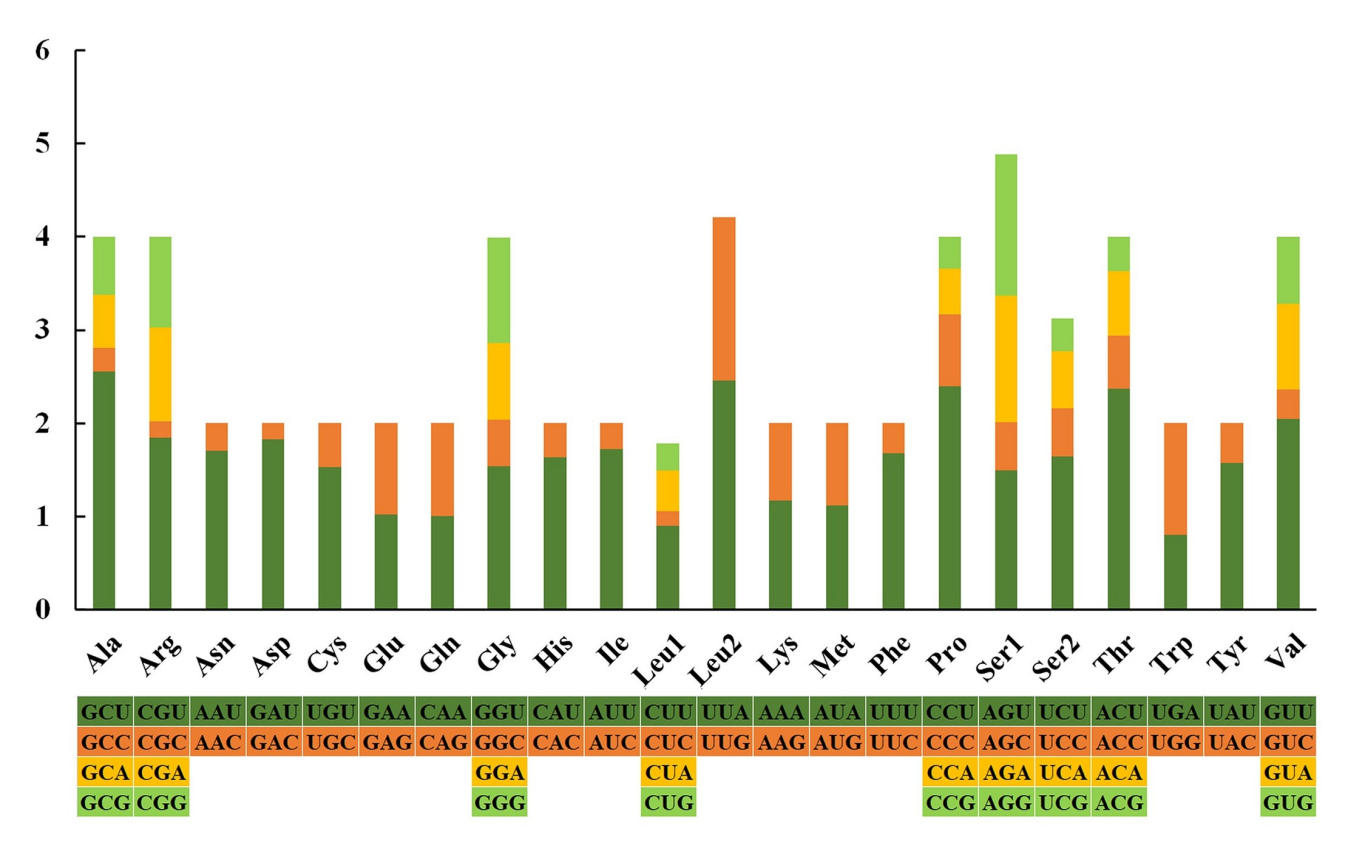
Relative synonymous codon usage (RSCU) in the mitogenomes of *S*. *minima*.

Most amino acids were used by either two or four in invertebrates, and only Leu and Ser were encoded by six and eight different codons, respectively [58]. The nucleotide relative synonymous codon usages (RSCUs) of *S*. *minima* are presented (Fig 3, Table 5). GCU (Ala), UUA (Leu2), CCU (Pro) and ACU (Thr) are the most frequently used codons, whereas CUC (Leu1), GAC (Asp) and CGC (Arg) are relatively scarce. As per the RSCU values, codons ending with an A or U were preferred, and the codons NNA and NNU were found in the majority.

**Table 5:** The codon number and relative synonymous codon usage in the mitochondrial genomes of *S*. *minima*.

Codon	Count	RSCU	Codon	Count	RSCU	Codon	Count	RSCU	Codon	Count	RSCU
UUU(F)	531	1.68	UCU(S)	121	1.64	UAU(Y)	204	1.57	UGU(C)	150	1.53
UUC(F)	102	0.32	UCC(S)	38	0.52	UAC(Y)	56	0.43	UGC(C)	46	0.47
UUA(L)	270	2.46	UCA(S)	45	0.61	UAA(*)	140	1.11	UGA(W)	86	0.80
UUG(L)	192	1.75	UCG(S)	26	0.35	UAG(*)	113	0.89	UGG(W)	128	1.20
CUU(L)	99	0.90	CCU(P)	78	2.40	CAU(H)	70	1.63	CGU(R)	40	1.84
CUC(L)	18	0.16	CCC(P)	25	0.77	CAC(H)	16	0.37	CGC(R)	4	0.18
CUA(L)	47	0.43	CCA(P)	16	0.49	CAA(Q)	35	1.00	CGA(R)	22	1.01
CUG(L)	32	0.29	CCG(P)	11	0.34	CAG(Q)	35	1.00	CGG(R)	21	0.97
AUU(I)	232	1.72	ACU(T)	96	2.37	AAU(N)	201	1.70	AGU(S)	110	1.49
AUC(I)	37	0.28	ACC(T)	23	0.57	AAC(N)	36	0.30	AGC(S)	38	0.52
AUA(M)	109	1.12	ACA(T)	28	0.69	AAA(K)	157	1.17	AGA(S)	100	1.36
AUG(M)	85	0.88	ACG(T)	15	0.37	AAG(K)	112	0.83	AGG(S)	111	1.51
GUU(V)	226	2.05	GCU(A)	107	2.55	GAU(D)	108	1.83	GGU(G)	181	1.54
GUC(V)	34	0.31	GCC(A)	11	0.26	GAC(D)	10	0.17	GGC(G)	59	0.50
GUA(V)	101	0.92	GCA(A)	24	0.57	GAA(E)	93	1.02	GGA(G)	96	0.82
GUG(V)	79	0.72	GCG(A)	26	0.62	GAG(E)	89	0.98	GGG(G)	133	1.13

**Fig 4:**
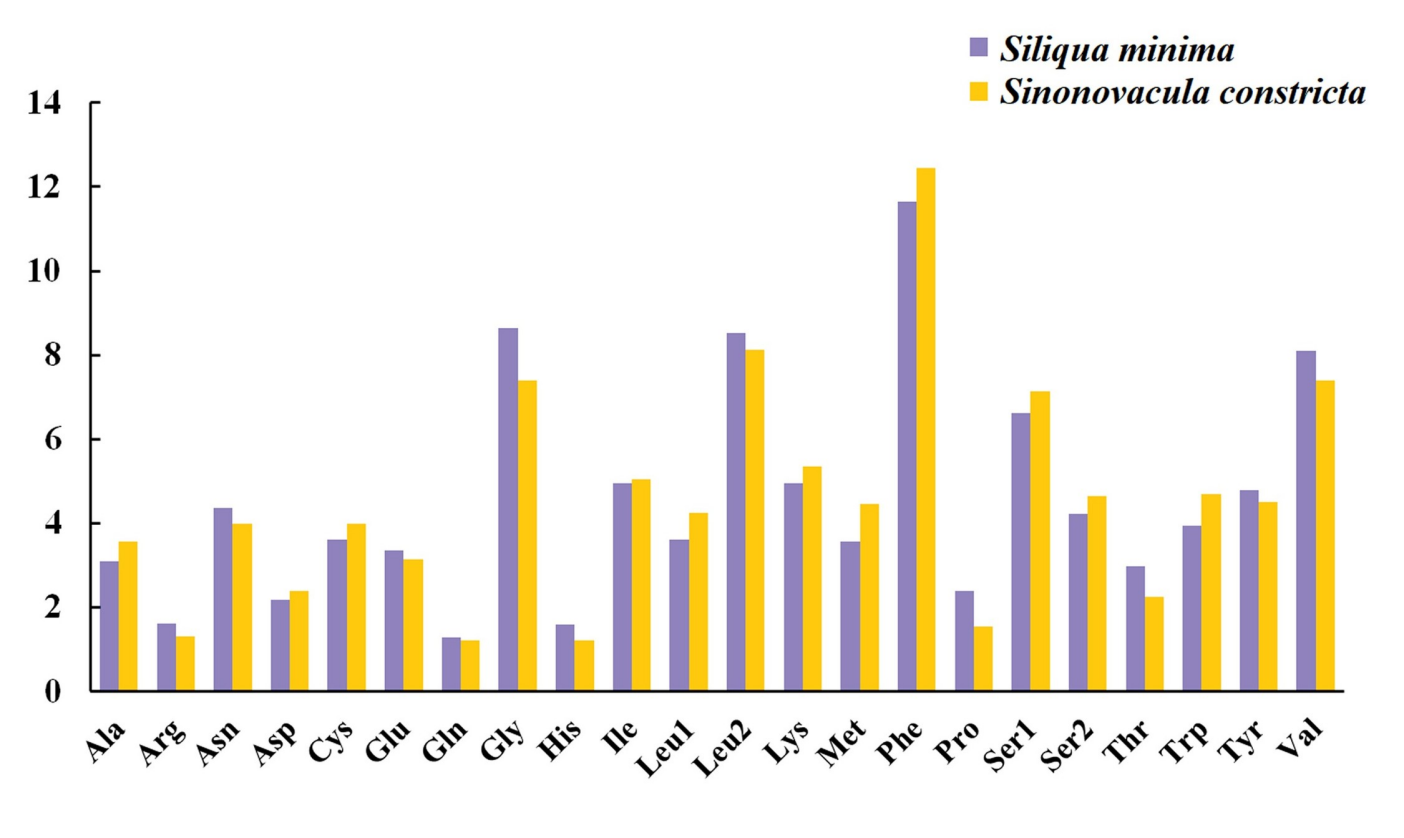
Amino acid composition of two Pharidae mitochondrial genomes.

In addition, we also compared the amino acid composition of two species of Pharidae (Fig 4). The four most frequent amino acids in the PCGs of *S*. *minima* were phenylalanine (11.66%), glycine (8.64%), leucine 2 (8.52%) and valine (8.10%), whose proportions were similar to those observed in *S*. *constricta*.

### Transfer and ribosomal RNA genes

The mitogenome of *S*. *minima* contained 22 tRNA genes varying in size from 63 to 76 bp, and each of them was unique and compatible with codon usage in invertebrate mitogenomes (Table 3). Two types of anticodons (TAG and TAA) determined leucine, and TCT and TGA determined serine. The average content of A+T in the entire tRNA was 66.12%. The AT-skew values were negative (-0.077), and GC-skew values were positive (0.256) (Table 2), indicating a bias towards Ts and Gs when horizontal alignment of 22 tRNAs was performed. In addition, the tRNAs of the mitogenomes of six Adapedonta species ranged from 62.67% (*Solen strictus*) to 68.01% (*Panopea abrupta*) (Table 4). We observed that the seven species of Adapedonta had negative AT skew and positive GC skew.

**Fig 5:**
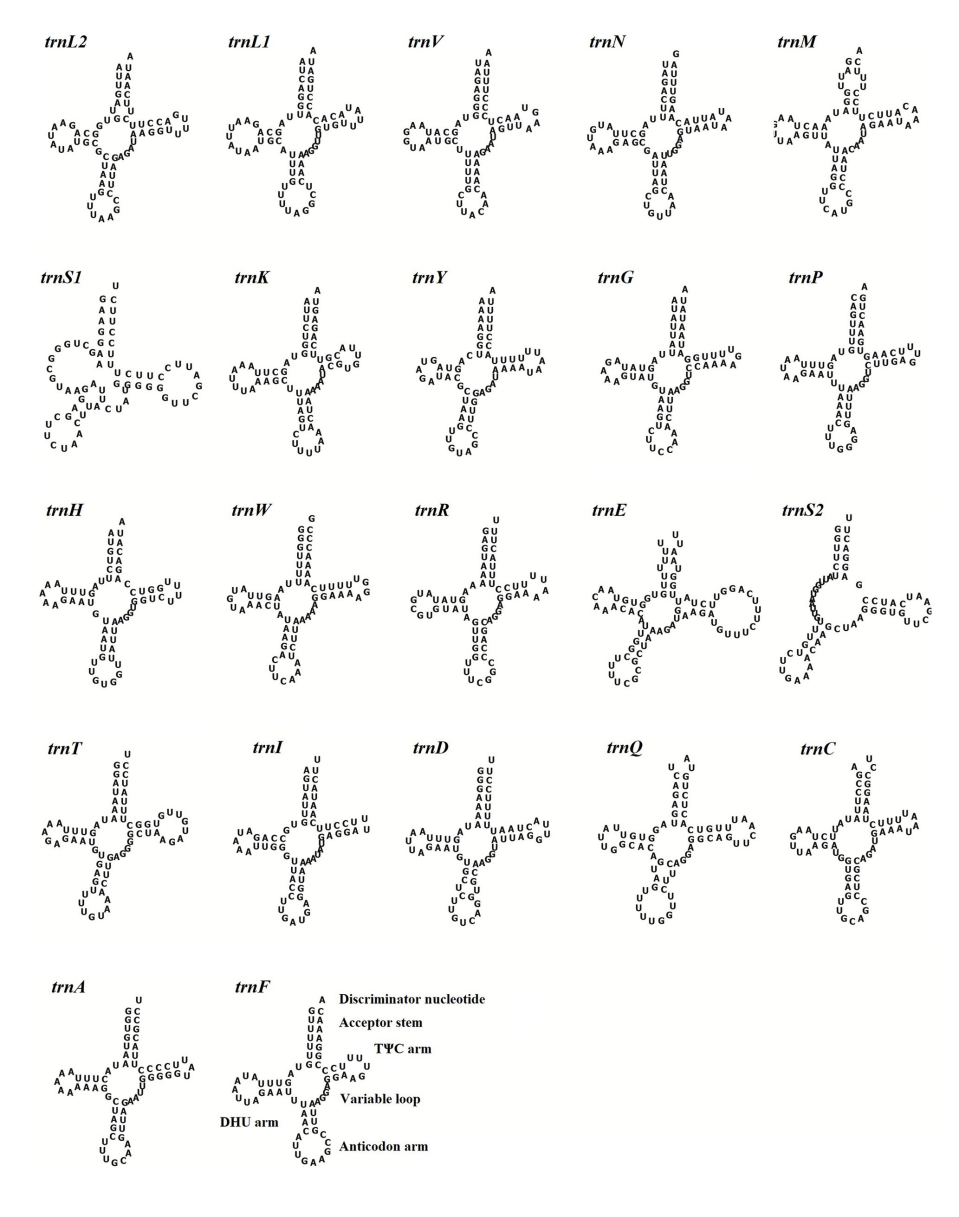
Secondary structure of the tRNA genes in the mitogenome of *S*. *minima*.

To understand the functional and structural characteristics of tRNAs, we predicted the secondary structures of 22 tRNAs of *S*. *minima* (Fig 5). Except for two *trnS* (TCT and TGA), which have the most different structures, all the tRNAs could fold into a typical cloverleaf secondary structure. Similar to other bivalves, *S*. *minima* has no discernible DHU (dihydrouridine) stem and loop and cannot be folded into a typical cloverleaf structure [59]. Otherwise, we found twenty tRNAs (except two *trnL*) with at least one G-T base pairs, which formed weak bonds. This base pairs can be corrected by post-transcriptional RNA editing mechanisms [60].

The 16S rRNA subunit (*rrnL*) and 12S rRNA subunit (*rrnS*) were 1,247 bp and 829 bp in size, respectively (Table 3). Both fragments were separated by *atp6* and *trnM* genes. The base composition of the rRNA genes was 34.97% A, 33.86% T, 18.98% G and 12.19% C, and the A+T content was 68.83% (Table 2). Notably, both AT-skew (0.016) and GC-skew (0.218) values of rRNAs were positive, which was different from other genes. This indicates that the A and G content is more prevalent in mitochondrial RNA genes.

### Gene arrangement

**Fig 6:**
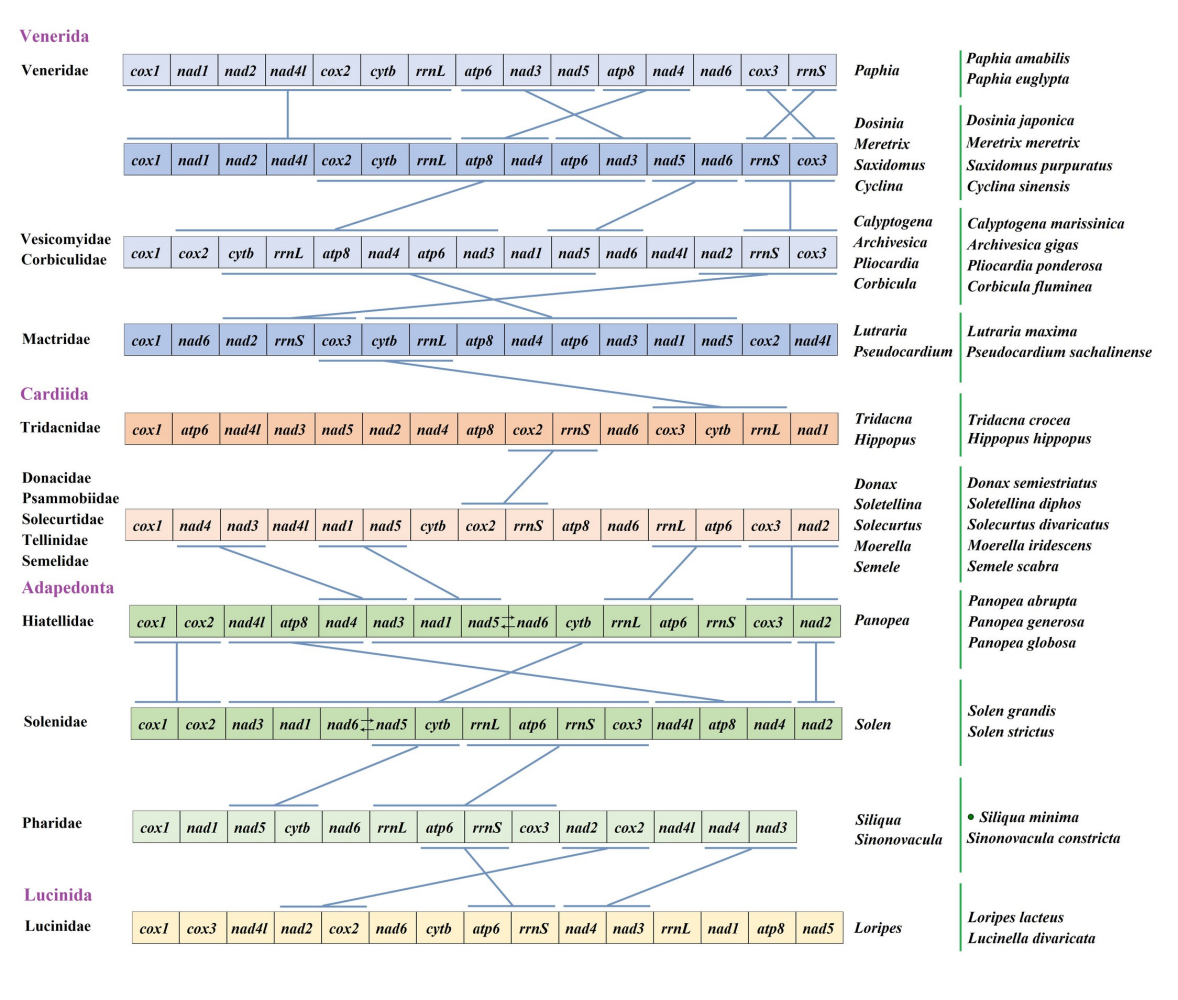
Linearized representation of the mitochondrial gene arrangement in Impardentia bivalves. The bars indicate identical gene blocks. Gene segments are not drawn to scale. The green dot indicates the specie of this study.

Gene order of the mitochondrial genome can be used to research the evolution of species. It can be used to investigate the ancestral lineage of phylogeny, and to establish the mechanism of gene replication, regulation and rearrangement. Bivalves of molluscs have highly variable mitochondrial gene sequences, and are the most mutated species in metazoa [61, 62]. In the study, we selected some species from four orders of the superorder Impardentia, Venerida, Cardiida, Adapedonta and Lucinida as representatives of bivalves to study mitochondrial gene rearrangement (Fig 6). Due to the great difference of gene sequence among bivalves, we excluded the tRNA genes and compared with them by 12 or 13 PCGs. Although their gene order are highly variable, we try to find out whether there are some shared gene blocks among bivalve species. The results showed that there was a mass of rearrangement in each order of bivalves, even if we deleted all tRNAs. The gene rearrangement analysis based on families or even genera is more appropriate. In addition, the sequence of genes in the four genera of *Dosinia*, *Meretrix*, *Saxidomus* and *Cyclina* were identical. Both of them contained *cox1-nad1-nad2-nad4l-cox2-cytb-rrnl* gene fragment. In addition, *atp6-nad3-nad5* and *atp8-nad4* were the same gene fragments, and *cox3* and *rrns* gene were interchanged. Compared with the families of Vesicomyidae and Corbiculidae, the gene order of the genus *Dosinia* and other four genera retained the overlength gene fragment of *cox2-cytb-rrnl-atp8-nad4-atp6-nad3*, as well as two small fragments of *nad5-nad6* and *rrns-cox3*. The sequence of two families Vesicomyidae and Corbiculidae, contains the same two gene blocks as that of the family Mactridae: *cytb-rrnl-atp8-nad4-atp6-nad3-nad1-nad5* and *nad2-rrns-cox3*. There is only one *cox3-cytb-rrnl* gene fragment in Mactridae, which is the same as Tridacnidae in Cardiida. Except for Tridacnidae, the gene sequences of the other five families in Cardiidae are identical. Donacidae, Psammobiidae, Solecurtidae, Tellinidae and Semelidae have the same arrangement as the family Tridacnidae. It also illustrates the family Tridacnidae is a very special family in Cardiidae. The gene arrangement of most families of the order Cardioidea is similar to that of the order Hiatellidae of the superorder Adapedonta in that there are four identical fragments *nad4-nad3*, *nad1-nad5-rrnl-atp6* and *cox3-nad2*. There are *nad2-cox1-cox2* and *nad4l-atp8-nad4* gene fragments in the same gene order between two families Hiatellidae and Solenidae, while *nad3-nad1-nad5-nad6-cytb-rrnl-atp6-rrns-cox3* gene fragment in the family Hiatellidae is almost the same as *nad3-nad1-nad6-nad5-cytb-rrnl-atp6-rrns-cox3* gene arrangement in the family Solenidae, only *nad5* and *nad6* genes in the reverse order. They are the two families with the closest gene arrangement in this study. The sequence of *nad5-cytb* and *rrnL-atp6-rrns-cox3* was the same as that of the family Pharidae. Moreover, species of Pharidae lack *atp8* gene, which is also common in bivalve species. Compared with the species of the family Lucinidae, there are only three identical gene blocks: *atp6-rrnS*, *nad2-cox2*, *nad4-nad3*. Through above analysis we can find that although the gene sequence among bivalve species is highly variable, the same gene arrangement is longer among the species with closer genetic relationship. However, the possibility of rearrangement of the contrast between the spanning orders is greater. It shows that there is a certain relationship between evolution and gene rearrangement, even in bivalve species with high rearrangement rate. However, in this study, there is a high degree of gene rearrangement, such as the family Tridacnidae. Thereby, the taxonomic evolution of species cannot be supported only by the study of gene sequence, but also needs the combination of phylogenetic reconstruction.

**Fig 7:**
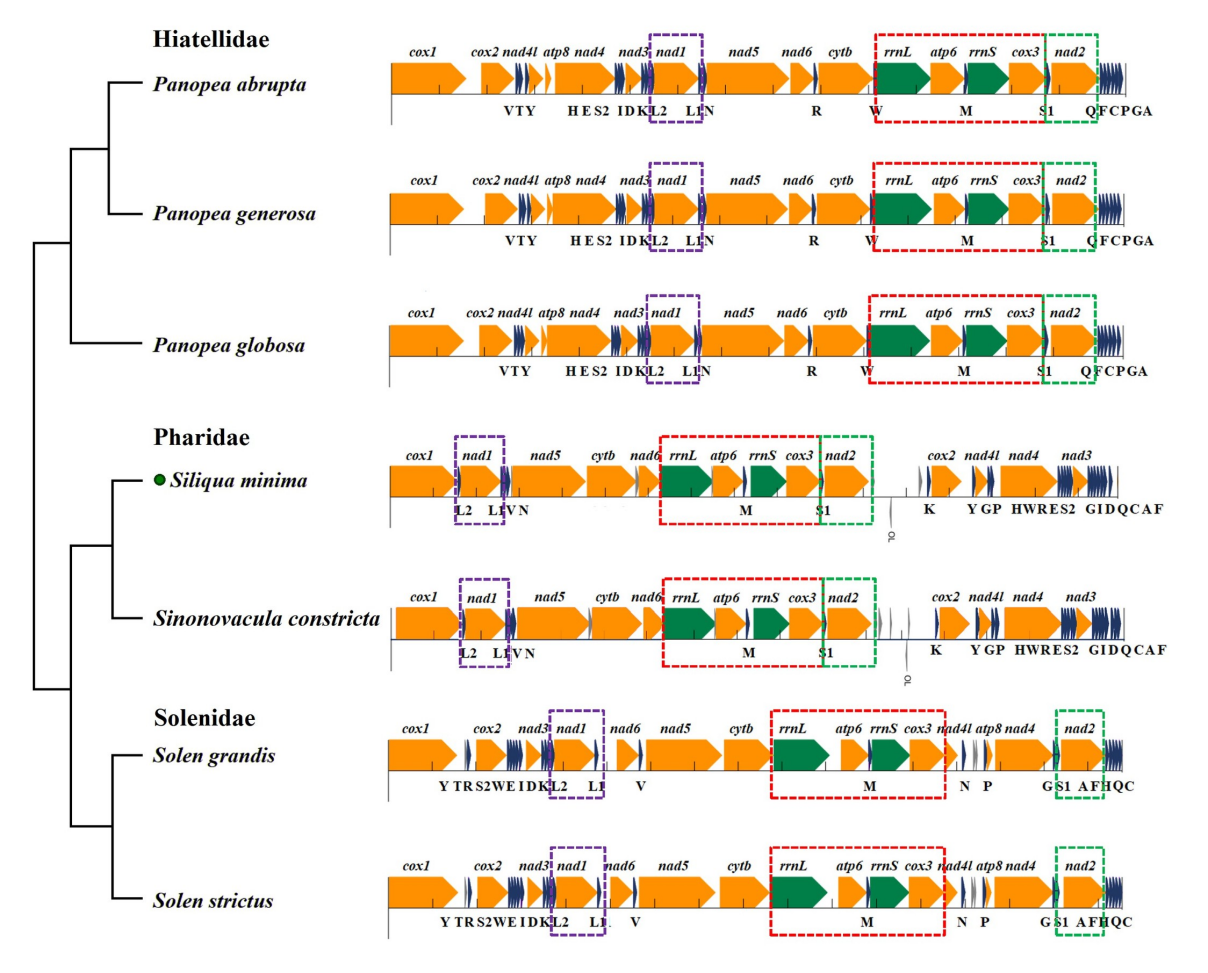
Comparison of mitochondrial gene rearrangements of the order Adapedonta. Gene segments are drawn to scale. Protein-coding, rRNA, and tRNA genes are shown in orange, green, and dark blue, respectively. Genes are oriented either to the right or to the left depending on whether they are encoded on the light or heavy strand. The dotted boxes in purple, red, and green represent fragments A, B, and C with the same gene order, respectively.

In addition, we further analyzed the species of the superorder Adapedonta using all the genes of mitogenome. Previously, gene rearrangement is rarely discussed separately in Adapedonta because of its extremely few whole mitogenome data. We propose the gene order analysis of three family species in Adapedonta first time, which can be used as a reliable phylogenetic marker for some bivalve lineages. The CR of Adapedonta species is typically only a small fragment or no obvious region. Nevertheless, we discovered that the CR was more than 1300 bp in family Pharidae. It is located between the *nad2* and *trnK* genes, and its order different from other Adapedonta species. In the mitogenome of Pharidae, a total of 10 genes, including 4 PCGs, 4 tRNAs and all rRNA gene are rearranged (Fig 7). As shown in Fig 7, three main gene blocks are described for Adapedonta, the gene arrangement in the family is relatively conservative, and only part of the difference comes from the base content. However, the gene rearrangement among the three families differed substantially, but some of the fragments were still retained. From the observation of seven species of Adapedonta, we can see that each species contained short fragments *trnL2-nad1-trnl1* (segment A), *rrnL-atp6-trnM-rrnS-cox3-nad2* (B) and *trnS1-nad2* (C), which were all behind *cox1* gene and in the same order. In the family Hiatellidae and Pharidae, fragments B and C are connected as a long fragment, which may be related to time of divergence.

### Phylogenetic relationships of Imparidentia

**Fig 8:**
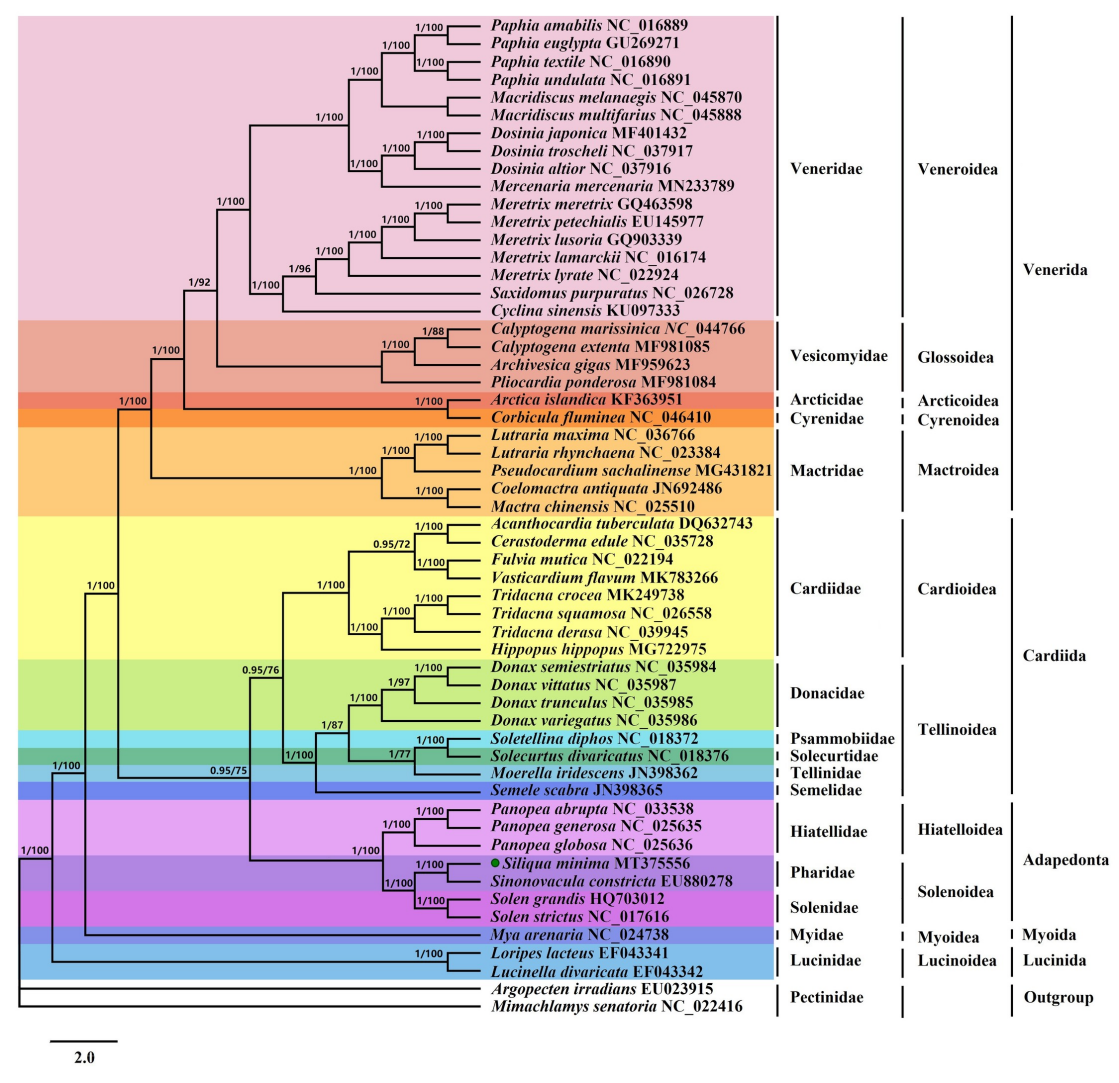
The phylogenetic tree for *S*. *minima* and other Bivalvia species based on 12 PCGs. Phylogenetic tree inferred using Bayesian inference (BI) and maximum likelihood (ML) methods, the PP value is in front of the node. the value on the left side of the slash is the posterior probabilities estimated by Bayesian tree, and the value on the right side is the maximum likelihood tree. The green dot indicates *S*. *minima* in this study.

To research the phylogenetic implications of the *S*. *minima* mitogenome in Imparidentia, we reconstructed the order-level phylogenetic tree. The phylogenetic trees based on Bayesian inference (BI) and maximum likelihood (ML) analyses of 12 PCGs of 54 species produced identical topologies (Fig 8). The tree topologies based on two methods were basically congruent and obtained high supports in the majority of nodes. The relationships among the five orders of Imparidentia involved herein were consistently recovered as (Venerida + (Cardiida + Adapedonta)) + Myoida + Lucinida, which is slightly different from the study of Fernandez-Perez et al. [46]. However, the results are consistent with the topological structure of phylogenetic tree constructed by using transcriptional data base on the morpho-anatomical by Lemer et al. [14]. In addition, this result is also basically consistent with phylogenetic tree constructed based on mitogenome by Yuan et al. [63], but we added a large number of mitochondrial genome data species on this basis to further explore the evolutionary relationship between bivalves. In our analysis, the evolutionary differences were mainly concentrated between three orders of Venerida, Cardiida and Adapedonta. We can find that Venerida is the first to branch out of the three orders, and its branching posterior probabilities and bootstrap values are higher than those of the previous studies with adapedont as the first branch. This confirmed that phylogenetic analysis based on our data is more effective. The analysis shows that two species of the order Lucinida were the outermost species of all bivalves and formed a single clade, i.e., Lucinida is monophyletic, in accordance with previous viewpoint [63]. Moreover, BI and ML recovered each the family Pharidae, Solenidae and Hiatellidae form a monophyletic assemblage with strong support. Both ML and BI analyses of two datasets supported the sister-group relationship of Pharidae and Solenidae species (Bayesian posterior probabilities (PP) = 1.00, and bootstrap values (BS) = 100), as previously reported [30]. In addition, the family Hiatellidae was placed as sister to Pharidae and Solenidae (PP = 1; BS = 100). The phylogenetic relationships between seven species in the order Adapedonta are (((*Panopea abrupta* + *Panopea generosa*) + *Panopea globose*) + *Hiatella arctica*) + ((*S*. *minima* + *S*. *constricta*) + (*Solen grandis* + *Solen strictus*)) (Fig 8). There has been controversy about the branch of *S*. *constricta* for a long time. It was once thought to be a member of the family Solenidae, and then it was classified into the family Tellinoidea by morphological identification and anatomical characteristics [64]. Yuan et al. used multiple PCGs to reconstruct the phylogenetic relationships and classified *S*. *constricta* within the family Solenidae [31]. In our study, it was obvious that *S*. *minima* and *S*. *constricta* of the family Pharidae form a new branch. The analysis of the mitochondrial genome in this study further strengthens the previous elevation of the order Adapedonta to the family level. In fact, at present, there has not been any special evolutionary research on the whole superorder species based on molecular data. Therefore, the study evolution and classification use molecular means base on morphological dissection, such as mitochondrial genome are still necessary to test the taxonomy of superorder Imparidentia.

## Conclusions

We sequenced and assembled the mitogenome of *S*. *mini*ma using next-generation sequencing, and the genome was 17,064 bp in length. The gene distribution was entirely presented on the heavy chain of the *S*. *mini*ma mitogenome. With the skewness of the *S*. *minima* mitogenome, except for the CR and rRNAs, most AT skews were negative; moreover, all GC skews were positive. In the tRNA secondary structure, only two *trnS* cannot be folded into a typical cloverleaf structure because they do not have a discernible DHU stem-loop. In the analysis of PCGs rearrangement of bivalve species, the gene sequence among species is highly variable, the more consistent of the same gene arrangement is longer among the species with closer genetic relationship. Furthermore, after analysis of homologous regions between the seven Adapedonta mitogenomes, it was concluded that the gene rearrangement among families is particularly obvious, while the gene rearrangement within families is relatively conservative. The phylogenetic trees constructed by ML and BI methods had the same branches. The results show that *S*. *minima* and *S*. *constricta* are the closest relatives and both belong to the family Pharidae. At present, the complete mitochondrial genome data of Pharidae are quite limited, and this study we reconstructed phylogenetic trees using the superorder Imparidentia, thus increases the understanding of the phylogeny of Pharidae.
